# Population Approaches to Prevention of Type 2 Diabetes

**DOI:** 10.1371/journal.pmed.1002080

**Published:** 2016-07-12

**Authors:** Martin White

**Affiliations:** Centre for Diet & Activity Research, MRC Epidemiology Unit, University of Cambridge, Cambridge, United Kingdom

## Abstract

Martin White argues that whole population interventions will be needed in addition to those targeted to people at high risk in order to respond to the global challenge of type 2 diabetes.

With the prevalence of Type 2 Diabetes Mellitus (T2DM) continuing to rise in most high-income, low-income, and middle-income countries [1], strategies to stem the emerging pandemic are urgently needed. To date, the best evidence for prevention of T2DM comes from randomized controlled trials of lifestyle interventions (e.g., to modify diet and physical activity and achieve weight loss) delivered to individuals at high risk, usually those with impaired glucose tolerance or prediabetes [2]. However, strategies that focus on changing individual behavior have limited reach due to the challenges of identifying at scale those in whom an intervention may be beneficial. Thus, while the behavioral interventions themselves have been shown to be meet acceptable cost-effectiveness thresholds, the cost-effectiveness of both identifying and intervening at the scale needed to achieve population impact remains less clear [3]. It is likely that, in the short term, such strategies will be unaffordable by all but the most affluent nations. Demonstration projects attempting to identify and deliver interventions to all those at high risk in the population (estimated to be >10% of adults in England, for example), such as the NHS Diabetes Prevention Programme, will therefore be watched with interest [4].

Behavioral interventions targeting individuals can also have variable effectiveness across population subgroups due to differential access, uptake, and compliance, resulting in lesser benefits in people from socioeconomically disadvantaged backgrounds [5]. Such interventions could therefore widen inequalities in incidence and prevalence of T2DM [6]. Such strategies will be of value to help stem the tide of new cases of T2DM but realistically need to be accompanied by more affordable and wider reaching population-level interventions to change diet and physical activity, reduce obesity, and thus reduce incidence of T2DM. Population intervention strategies will be particularly important for low-income and middle-income countries, for whom individual-level interventions may be unaffordable [7]. Such approaches have been advocated by WHO, which calls for “multisectoral action that simultaneously addresses different sectors that contribute to the production, distribution, and marketing of food, while concurrently shaping an environment that facilitates and promotes adequate levels of physical activity” [8].

Population approaches to prevention aim to reduce key risk factors for T2DM in the whole population, irrespective of individual level of risk. They achieve this by bringing about small changes in risk factor levels in the whole population, resulting in a shift in the population distribution of risk. Such shifts in the distribution of disease risk can theoretically lead to substantial population benefit [9], and the principle has been well demonstrated in relation to, for example, salt consumption and blood pressure [10]. Such approaches may not be entirely without harm, however. For example, not everyone in a population needs to reduce weight, reduce blood pressure, or become more active [11]. But given the small expected change in risk at an individual level, and their delivery in a societal context, population interventions are generally considered safe [9].

There are potential concerns also about the differential effectiveness of some population interventions. Not all population-level interventions are likely to have the same impact, and some may be less equitable than others. This is a particular concern for interventions that require a higher level of engagement by individuals, such as food labelling, which demands literacy and numeracy as well as an ability to process and apply the information presented in making healthy food choices, often from a bewildering array of available products [12]. Interventions requiring low levels of individual engagement, such as regulation of TV advertising of unhealthy foods, may be more equitable and offer greater overall health impacts. However, such policy interventions need to be formulated to ensure population benefit and be rigorously evaluated [13].

Simulation studies have modelled the potential impacts on T2DM incidence of small changes in risk factors for T2DM, such as physical activity at a population level, demonstrating significant potential benefits [14]. The challenge is to develop and deliver interventions that can bring about such small changes across the whole population cost-effectively. Is this achievable and, if so, how?

Population level interventions can be delivered via a range of modalities. Each is underpinned by a policy measure (e.g., a law or voluntary agreement) and involves a mechanism to achieve change in risk exposure. The mechanisms are diverse and include, for example, reformulation of foods (e.g., to reduce sugar content), provision of information (e.g., food labelling), fiscal measures (e.g., taxes on less healthy food products), or structural and environmental measures (e.g., new infrastructure for active commuting, such as cycle lanes). These can usefully be categorized as technology, education, or resource-based and sometimes involve combinations of these modalities (Table 1).

**Table 1:** Potential examples of population level interventions to change diet and physical activity behaviors, by principal modality.

	Diet	Activity
Policies	Regulation of TV advertising of foods high in salt, fat, and sugar	Curriculum policy concerning physical education in schools
	Voluntary agreements with the food industry	Urban transport policy (public transport, cycling, car parking)
	Fiscal policy governing taxation of foods	Fiscal policy (e.g., Value Added Tax on sports clothes, equipment, bicycles)
	Planning policy governing the siting and opening of take-away food outlets	Workplace activity policies (e.g., work time credits for activity breaks)
Education	Informative labelling of packaged foods and menus	Informative prompts to use stairs instead of lifts/escalators
	Mass communication/social marketing campaigns on television, radio, or print media	Mass communication/social marketing campaigns on television, radio, or print media
Technologies	Reformulation of processed foods	Infrastructure to support active commuting (e.g., cycle lanes, secure bicycle parking, widely accessible bicycle rental schemes)
	Mobile applications to support dietary change	Mobile applications and personal technologies to support an active lifestyle
Resources	Taxes on less healthy food products (e.g., sugar-sweetened beverages)	Urban congestion charging, limiting car use
	Price reductions on healthier food products (e.g., vegetables, whole grains)	Subsidies for physical activity at local leisure centers

Many such interventions have been implemented with apparent success [15–17]. While the primary aim has usually not been T2DM prevention specifically, their contributions to this goal may be considerable. For example, an evaluation of a new guided busway in Cambridgeshire, United Kingdom, found that it resulted in an increase in active commuting; participants living 4 km from the busway were one-third more likely to have increased their cycle commuting between 2009 and 2012 than those living 9 km away, reporting a mean increase of 87 minutes of cycling per week [18]. Similarly, early evaluation of the tax on sugar-sweetened beverages in Mexico suggests a decrease in purchases of taxed beverages and an increase in purchases of untaxed beverages [19]. Some evidence suggests that exposure to favorable diet and physical activity environments, such as healthy food provision and physical activity resources at neighborhood level, may have measureable effects on T2DM incidence [20]. In these intervention examples, it is possible that planned environmental changes will impact total activity level, or total sugar and energy consumption, with potentially important consequences for body mass, glucose regulation, and associated health outcomes at a population level.

Evaluating the impact of population-level preventive interventions on health outcomes is a challenging long-term goal that needs investment in routine data systems, data linkage, and new paradigms of experimental research that are able to capture impacts across a range of potential outcomes at a system level, taking into account their contextual complexity. The challenges of such evaluations have been helped considerably by methodological developments in recent years, which have been given prominence by guidance on evaluation of natural experiments from the UK Medical Research Council [21]. Nevertheless, evaluating population interventions is likely to continue to prove challenging, especially in countries with poorly developed routine heath information systems.

While rigorous science is needed to build the evidence base for population interventions, this alone will not be sufficient to ensure that the range of interventions needed to reshape population risk of T2DM is delivered by countries and regions. Such interventions are delivered in real world contexts by policy makers at local and national levels outside the control of researchers. Scientific evidence is just one of a number of inputs to the policy-making process, and political and economic considerations are usually of equal or greater importance [22,23]. Research thus needs to focus on these too, identifying, in particular, the economic case for such interventions, not just in terms of cost-savings to health systems, but also in terms of the wider benefits to society from reduced morbidity, including improved employment prospects and economic productivity.

The global challenges of obesity and T2DM are inextricably linked, and their scale and economic impact demand global action from political leaders. Interventions need to include both those targeted to people at high risk (to address the existing burden of prediabetes) and those aimed at whole populations (to reverse current upward trends). Solutions will need to be sensitive to context in low-income, middle-income, and high-income countries. Population interventions may prove the most viable options in resource-poor nations [7]. Bold policy decisions will need to be made to avert the economic and health crises that are the likely consequences of current trends in obesity and T2DM, since we cannot wait for perfect evidence [24].
